# BAC library resources for map-based cloning and physical map construction in barley (*Hordeum vulgare *L.)

**DOI:** 10.1186/1471-2164-12-247

**Published:** 2011-05-19

**Authors:** Daniela Schulte, Ruvini Ariyadasa, Bujun Shi, Delphine Fleury, Chris Saski, Michael Atkins, Pieter deJong, Cheng-Cang Wu, Andreas Graner, Peter Langridge, Nils Stein

**Affiliations:** 1 Leibniz Institute of Plant Genetics and Crop Plant Research (IPK), Corrensstr. 3, 06466 Gatersleben, Germany; 2 Australian Centre of Plant Functional Genomics, University of Adelaide, PMB 1 Glen Osmond SA 5064, Australia; 3 Clemson University Genomics Institute (CUGI), 51 New Cherry St. BRC 310, Clemson, SC 29634, USA; 4 BACPAC Resources, Children's Hospital Oakland, 747 52nd St. Oakland, CA 94609, USA; 5 Lucigen Corporation, 2120 West Greenview Dr., Middleton, WI 53562, USA; 6 KWS SAAT AG, Grimsehlstr. 31, 37555 Einbeck, Germany

## Abstract

**Background**

Although second generation sequencing (2GS) technologies allow re-sequencing of previously gold-standard-sequenced genomes, whole genome shotgun sequencing and *de novo *assembly of large and complex eukaryotic genomes is still difficult. Availability of a genome-wide physical map is therefore still a prerequisite for whole genome sequencing for genomes like barley. To start such an endeavor, large insert genomic libraries, i.e. Bacterial Artificial Chromosome (BAC) libraries, which are unbiased and representing deep haploid genome coverage, need to be ready in place.

**Result**

Five new BAC libraries were constructed for barley (*Hordeum vulgare *L.) cultivar Morex. These libraries were constructed in different cloning sites (*Hind*III, *EcoR*I, *Mbo*I and *BstX*I) of the respective vectors. In order to enhance unbiased genome representation and to minimize the number of gaps between BAC contigs, which are often due to uneven distribution of restriction sites, a mechanically sheared library was also generated. The new BAC libraries were fully characterized in depth by scrutinizing the major quality parameters such as average insert size, degree of contamination (plate wide, neighboring, and chloroplast), empty wells and off-scale clones (clones with <30 or >250 fragments). Additionally a set of gene-based probes were hybridized to high density BAC filters and showed that genome coverage of each library is between 2.4 and 6.6 X.

**Conclusion**

BAC libraries representing >20 haploid genomes are available as a new resource to the barley research community. Systematic utilization of these libraries in high-throughput BAC fingerprinting should allow developing a genome-wide physical map for the barley genome, which will be instrumental for map-based gene isolation and genome sequencing.

## Background

Bacterial artificial chromosome (BAC) libraries are the large DNA insert libraries of choice and an indispensible tool for map based cloning, physical mapping, molecular cytogenetics, comparative genomics and genome sequencing. In contrary to their name, BACs are not artificial chromosomes *per se*, but rather are artificial bacterial F factor derived constructs [1]. Although BACs could carry inserts approaching 500 Kb in length, insert sizes are typically between 80 and 200 Kb in plants [2-8]. Cloning into BAC vectors rarely leads to chimeric or rearranged inserts [9-13] due to the presence of F factor genes (*parA *and *parB*) that prevent bacteria from maintaining more than one BAC simultaneously. An additional advantage of BAC clones is their easy manipulation and propagation compared to viral or yeast-vector based systems [14-16]. Consequently BACs have supplanted YACs as the dominant vector for large insert libraries and have been abundantly used in large-scale physical mapping projects [17-21]. Physical maps are pivotal for whole genome sequencing strategies of large and complex genomes. They are also instrumental to the scientific community for gene isolation [21,22]. A genome-wide physical map of the maize genome was built as a basis for genome sequencing [23]. A chromosome-specific BAC library strategy has been adopted for bread wheat (*Triticum aestivum *L.) to cope with the presence of three highly related homoeologous genomes [20,24]. For the diploid barley (*Hordeum vulgare *L.) genome, the International Barley genome Sequencing Consortium (IBSC) [25] set out to develop a deep coverage well ordered whole genome physical map [21] as a platform for trait isolation and genome sequencing.

Large insert genomic libraries which are unbiased and representing few folds of the haploid genome are a key factor for successful generation of a physical map [18]. BAC libraries with very large inserts can be readily constructed with the partial digestion method; however, unbiased large-insert BAC libraries may be built only from mechanically sheared high molecular weight genomic DNA in order to generate random fragments across the genome [26]. A synergistic approach of combining libraries created by different methods will help in reducing gaps in the physical map that may result from uneven distribution of restriction sites of the employed restriction endonucleases. BAC maps which provided the basis for genome sequencing [18,23,27,28] benefited immensely by combining multiple libraries.

Until recently, four BAC libraries of barley have been published. One was derived from a North American six-rowed malting variety 'Morex' with 313,344 gridded clones (6.3-fold haploid genome coverage [29]. Two further libraries have been reported for the cultivars *Haruna Nijo *[30] and *Cebada Capa *[31]. More recently a fourth library was constructed from a doubled haploid barley line CS134 derived from a cross between the Australian malting variety 'Clipper' and the Algerian landrace Sahara 3771 [32]. It is noteworthy that all these libraries have been extensively used for characterizing and isolating genomic regions of interest [31-34]. However, for barley in general and Morex in particular, the depth of available resources (haploid genome coverage, diverse restriction enzymes, etc.) was far too shallow to provide raw material for a genome wide physical map.

Here, we report on the development of five BAC libraries derived from cultivar 'Morex', which has been selected by IBSC as the reference genotype for genome sequencing. The aim of IBSC was to generate BAC resources by different complementing approaches in order to reach sufficient and synergistic genome coverage for a representative whole genome physical mapping. The new libraries which are publicly available are described here.

## Methods

### Plant material

Barley seeds of the progeny "Morex 2003#9" kindly provided by Professor Patrick Hayes at Oregon State University, USA, was used in the library construction. About 200-400 seeds were grown under green house conditions. For the isolation of nuclei for high molecular weight (HMW) DNA preparation, etiolated leaves were harvested from 4-6 weeks old plants.

### Construction of BAC libraries from partially restricted DNA

The libraries HVVMRXALLhB, HVVMRXALLhC, HVVMRXALLmA and HVVMRXALLeA were constructed from partially digested and size fractionated high molecular weight (HMW) DNA following previously published procedures [32,35,36] (Table 1). In brief, purified DNA (150 Kb-500 Kb) was ligated to the linearized and dephosphorylated vector. The ligation mixture was transformed into competent cells by electroporation. White recombinant colonies were selected on LB plates containing 12.5 - 20 μg/μl chloramphenicol, 90 μg/μl 5-Bromo-4-Chloro-3-Indolyl-Beta-D-Galacto-Pyranoside (X-Gal), 90 μg/μl Isopropyl-beta-D-Thiogalactopyranoside (IPTG), and picked robotically.

**Table 1:** New BAC libraries available from cultivar Morex.

BAC-library^1,2^	No. of clones	Average insert size (Kb)	Genome coverage	Cloning strategy	Vector	*E. coli *strain
HVVMRXALLhB^3^	115,200	93	2.4	*Hin*dIII, partial	pIndigo BAC5	DH10B
HVVMRXALLhC^3^	153,600	114	3.4	*Hin*dIII, partial	pIndigo BAC5	DH10B
HVVMRXALLeA^4^	147,456	126	3.7	*Eco*RI, partial	pIndigo BAC536	DH10B
HVVMRXALLmA^5^	202,752	143	6	*Mbo*I, partial	pTAR BAC1.3	DH10B
HVVMRXALLrA^6^	253,440	92	4.7	Mech. Sheared	pSMRT BAC	DH10B

^1^Naming follows an agreed nomenclature for *Triticeae *BAC libraries: three letters species code (Hordeum vulgare ss. vulgare), three letters cultivar code (Morex), three letters genome representation (i.e. ALL = whole genome, 1HS = short arm chromosome 1H), one letter enzyme (i.e. h = *Hind*III, e = *EcoR*I, m = *Mbo*I, r = Random/Mechanically sheared), one letter library identifier in case of multiple libraries per enzyme; ^2^all libraries are accessible via http://cnrgv.toulouse.inra.fr/en/library/barley; libraries were produced at: ^3^Australian Centre of Plant Functional Genomics, ^4^Clemson University Genomics Institute, ^5^Childrens Hospital Oakland, ^6^Lucigen corporation.

Recombinant clones were transferred into individual wells of microtiter plates, grown and then stored at -80°C. Library HVVMRXALLrA (Table 1) was produced from mechanically sheared DNA as previously described [35,37]. Briefly, the HMW DNA (at least 20 μg) plugs were melted at 75°C, mechanically sheared and size fractionated on a Clamped Homogeneous Electrical Field (CHEF) gel. The large DNA fragments were then subjected to end-repairing and polished by Lucigen's DNA terminator kit. The "polished" blunt-ended DNA was ligated to a *BstX*I linker to create 5' protruding, non-complementary (CACA) ends according to manufacturer's instruction. The linker-ligated large DNA fragments were size-fractionated by pulsed-field gel electrophoresis, permitting the simultaneous removal of excess free linker and isolation of the sized genomic fragments (100~200 Kb). The size-fractionated DNA retains 5' extending ends, which can be ligated to the complementary 5' (TGTG) ends of the *BstX*I digested pSMART-BAC cloning vector. Electroporation of the BAC ligation mixture was performed and BAC clones were randomly picked for BAC DNA preparation, *Not*I digestion and insert size check. The final BAC library was assembled from transformed ligations that delivered clones with average insert size of 100 Kb or larger.

### DNA isolation of plasmid-DNA

BAC-Plasmid DNA was isolated in a semi-automated approach utilizing NucleoSpin 96 Flash kit (Macherey&Nagel, Germany). Bacterial cultures were inoculated with a 96 pin replicator directly from glycerol stocks (384-well storage plates) into deep-well plates (96-well; Macherey&Nagel, Germany) containing 1.3 ml/well of either 2× YT-medium [38] (HVVMRXALLhA, HVVMRXALLeA, HVVMRXALLhB, HVVMRXALLhC), or 2x LB-medium [38] (HVVMRXALLmA, HVVMRXALLrA), respectively. In order to introduce positive and negative controls to each culture plate, two pins were removed from the replicator. After inoculating 94 clones, a positive control clone (HVVMRXALLhA0318G23) was introduced manually to the well H01 whereas well H12 was not inoculated with any clone thus serving as a negative control to monitor cross contaminations from the inoculation procedure. The cultures were grown at 37°C for 16-22 h agitated at 250 rpm on an orbital shaker (Infors AG, Switzerland). Cells were harvested by centrifugation (Heraeus Multifuge 35-R, thermo electron cooperation) of culture plates at 2,500 rpm for 15 min. The BAC DNA was isolated according to the manufacturer's instructions and eventually suspended in 50 μl molecular de-ionized water.

### High information content fingerprinting

High information content fingerprinting (HICF) was essentially performed according to published procedures [39]. In brief, 42 μl of BAC DNA was inoculated with 8 μl of a restriction mix consisting of two units of *Bam*HI, *Eco*RI, *Xba*I, *Xho*I and *Hae*III (New England Biolabs NEB, Germany), 1× NEB Buffer 2, 1× BSA, 0.5 μg DNAase-free RNase A and 0.02% beta-mercaptoethanol for 3 h at 37°C. Ten μl of restricted product was incubated with the labeling cocktail containing 0.3 μl SNaPshot Multiplex Reaction Mix (Applied Biosystems, Germany), 2 μl NEB-Buffer 2, 2.5 μl 100 mM Tris/HCl (pH 9.0) and 5.2 μl de-ionized water (1 h at 65°C).

Fragmented and labeled DNA was precipitated by adding 5 μl 2.5 M sodium-acetate and 100 μl 99% ethanol (-20°C) followed by incubation at -80°C for 15 min. DNA was collected by centrifugation at 4,200 rpm for 30 min. The pellet was washed with 100 μl 70% ethanol, air dried and re-suspended in 9.8 μl Hi-DiTM Formamide and 0.2 μl GS1200LIZ size standard (Applied Biosystems, USA). The samples were denatured at 95°C for 5 min before loading to the capillary sequencer ABI3730xl (Applied Biosystems). The capillary electrophoresis was performed on 50 cm capillary arrays using ABI's default run module for 108 min 3730 running-buffer with EDTA and 3730 POP-7TM polymer (Applied Biosystems, Germany).

### Analysis of fingerprinting data

Peak areas, peak heights and fragment sizes of each BAC fingerprint profile were collected by ABI's data collection program. The raw data was assessed for sizing quality using GeneMapper v4.0 (Applied Biosystems, Germany). An electronic fingerprint was assigned with the software FPPipeliner v1.0 and further analyzed for organelle contamination, neighboring, and plate-wide contamination with FPMiner (BioinforSoft LLC, USA).

The software was also used for automatic elimination of vector borne fragments in all fingerprint profiles. Furthermore FPminer was used to distinguish the peaks between true fragments and those originating from background noise or 'snapshot' artifacts. The edited profiles were exported as sizes files in order to perform contig assembly with the assembly program FPC V9.0 [40].

### Insert size determination

For insert size determination 10 μl of isolated BAC-plasmid-DNA was digested for 4 h at 37°C with 5 Units *Not*I (Fermentas, Germany) in 1× Buffer 3 containing 1× BSA. The digested DNA was separated together and sized with a low range Pulsed Field Gel Electrophoresis (PFGE) marker (New England Biolabs) by PFGE (CHEF DRIII, Biorad, Germany) on 1% agarose gels in 0.5 × TBE; (14°C, 6.0 V/cm, angle = 120, initial switch time 5 sec, final switch time 15 sec, run time = 16 h and ramping = linear).

### Screening of BAC libraries

Screenings of all BAC libraries were performed on high density colony filters (see additional file 1). Hybridizations were performed as described previously [38]. Membranes were prehybridized with 6× SSC, 5× Denhardt and 1 mg of denatured Salmon-sperm (Stratagene, USA) for 3 h at 68°C. Approximately 25 ng of probe was labeled separately with Megaprime kit (GE Healthcare, USA) and purified with Centrisep™ Columns (Applied Biosystems, Germany) according to manufacturer's instructions. Prior to hybridizations the probes were pooled and denatured at 95°C for 5 min followed by snap cooling on ice for another 5 min. Hybridizations were performed for at least 16 h at 68°C. Subsequently, membranes were washed once in buffer 1 (2× SSC, 0.1% SDS) followed by buffer 2 (1× SSC, 0.1% SDS) each at 68°C for 30 min. The filters were exposed for 4 h on imaging plates (Fuji film, Germany) and scanned on a FLA-3000 Phosphoimager (Fuji film, Germany). Positive BAC coordinates were identified with the software HDRF (Incogen, USA) and confirmed either by colony PCR or via colony hybridization [38]. Barley probes were designed from EST-sequences originating from the HarvEST Assembly 35 [41] (see additional file 2). Additionally 17 wheat probes were hybridized to the filter set of library HVVMRXALLhC. Prior to hybridization, quality and the copy number of the wheat probes was evaluated on Southern blots containing DNA from wheat nulli-tetrasomic lines as described by Pallotta et al., 2000 [42].

### Ordering of BAC libraries and filters

The library HVVMRXALLhA was published before [29] and can be obtained from Clemson University Genomics Institute (CUGI) [43]. The libraries HVVMRXALLhB, HVVRMXALLeA, HVVMRXALLmA, and HVVMRXALLrA are available from the Centre National de Ressources Génomiques Végétales (CNRGV) [44]. The high density colony arrays are available for the respective BAC libraries from the two resources centers CUGI and CNRGV (see additional file 1). The HVVRMXALLeA library and its filters can also be ordered from CUGI [43]. Library HVVMRXALLhC and filter sets were constructed and screened at Australian Center of Plant Functional Genomics (ACPFG, Adelaide, Australia).

## Results and Discussion

BAC libraries are the foundation for map-based gene isolation and physical map construction for un-sequenced genomes. Such physical maps were instrumental for sequencing several important plant genomes like rice [45] and maize [6,46]. Even for smaller plant genomes that are principally amenable for whole genome shotgun sequencing (WGS), the additional support provided by a physical map greatly facilitated ordering of the sequence contigs into scaffolds or super-scaffolds [47-49]. In crop species with genomes larger than 5 Gbp like barley, access to a physical map was proposed to be crucial to endeavor whole genome sequencing [21]. Additionally, a physical map would facilitate tremendously the isolation of genes underlying important traits in the Triticeae species. The systematic and high-throughput characterization of libraries is a pre-requisite for developing physical maps.

### Diverse BAC libraries to ensure high genome representation

Five new BAC-libraries of barley cultivar Morex were constructed (see Table 1). Of those, four libraries were constructed from partially digested high-molecular weight (HMW) DNA. Two of the libraries (HVVMRXALLhB and HVVMRXALLhC) were derived by partial digestion with enzyme *Hin*dIII, whereas the remaining was derived from partial digest with *Eco*RI (HVVMRXALLeA) or *Mbo*I (HVVMRXALLmA) (Table 1), respectively. The enzymes *Hind*III and *Eco*RI recognize 6 bp palindromes whereas *Mbo*I cleaves at a 4 bp palindromic site. A fifth library was obtained from cloning mechanically sheared HMW DNA.

The rationale behind constructing independent BAC libraries by partial digestion with different restriction endonucleases is that the frequency of occurrence of a specific palindrome in the DNA sequence is a function of the bp-composition of a species genome and of the recognition site [28]. Selecting multiple enzymes with a different recognition sequence would limit the risk of under-representation of specific regions of the genome of interest in the resulting BAC map [50]. The strategy of combining different BAC libraries was previously followed in other physical mapping projects such as soybean, bovine, *Brassica rapa *and maize [23,50-52]. To further overcome the bias of under-represented regions in libraries made of partially digested DNA, one BAC library was generated from mechanically sheared DNA (HVVMRXALLrA, Table 1). As described for rice [53], gaps in physical maps may occur because of non-random distribution of cloning sites, unstable DNA structures in *E. coli *hosts like Z-DNA, long inverted terminal repeats and AT-rich sequences [54,55]. Closure of such gaps is crucial to reach completion of a physical map. For example random sheared fosmid clones enabled the filling of gaps in the rice physical map in regions where there was no restriction site for BAC libraries [53]. Interestingly these clones contained genes of agronomical importance. Furthermore, its demonstrated that megabase-size DNA lacking any restriction site can be mechanically sheared as well as the DNA from other genomic regions [37] resulting in evenly distributed BACs across the genome. Therefore such libraries hold a high potential of gap closure. For example the random sheared BACs of the *Arabidopsis thaliana *genome played a crucial role in centrometric gap closure of the *Arabidopsis *physical map [26]. Therefore, generating a single random sheared BAC library with sufficient genomic coverage provides an important BAC resource and a complementing tool for a generic physical map of the barley genome.

### BAC libraries provide 25-fold genome coverage

**Figure 1:**
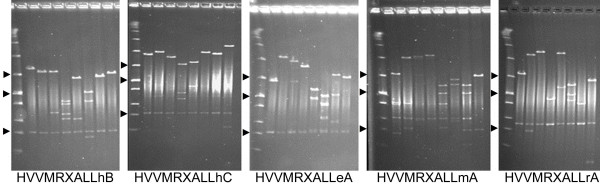
**Insert size estimation of barley BAC clones by pulsed field gel electrophoresis (PFGE)**. A sample set of 8 clones is visualized for all five new libraries. Each set of eight clones is preceeded by a lane showing low range PFG marker (New England Biolabs).Arrowheads indicate in each panel the position of the 97, 48.5 and 6.55 kb fragments of the PFG marker.

**Figure 2:**
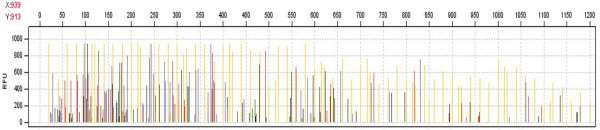
**Example of a BAC clone characterized by High Information Content Fingerprinting (HICF)**. After restriction digestion, SNaPShot labeling and separation of labeled fragments (red, blue, green, black) the sample was loaded together with the size standard GS1200LIZ (orange). Image is a screenshot taken from FPMiner 2.0 software

Genome representation of a given BAC library is important as it allows predicting the potential to find any given gene at least on a single BAC clone. Genome representation is a function of the overall number of unique clones and their respective insert sizes. Insert sizes of the clones were determined by *Not*I digestion and Pulsed Field Gel Electrophoresis (PFGE) of about 1330 clones (Table 2, Figure 1) as well as by HICF of ~10,000 BACs for each library (Figure 2).

**Table 2:** Result of insert size determination after *Not*I-restriction and PFGE analysis.

BAC-library	No of clones for NotI restriction	Average insert size (Kb)	No. of clones for HICF	Average fragment number (after HICF)*
HVVMRXALLhA	n.d.	106	10,435	87.9
HVVMRXALLhB	175	93	10,346	96.7
HVVMRXALLhC	181	114	10,279	101.1
HVVMRXALLeA	304	126	10,414	104.1
HVVMRXALLmA	303	143	10,685	123.8
HVVMRXALLrA	366	92	10,679	87.4

In addition the average fragment number after HICF is listed for all BAC libraries based on a random set of investigated clones. * Size standard = GS1200LIZ (Applied Biosystems); n.d. = not determined

**Figure 3:**
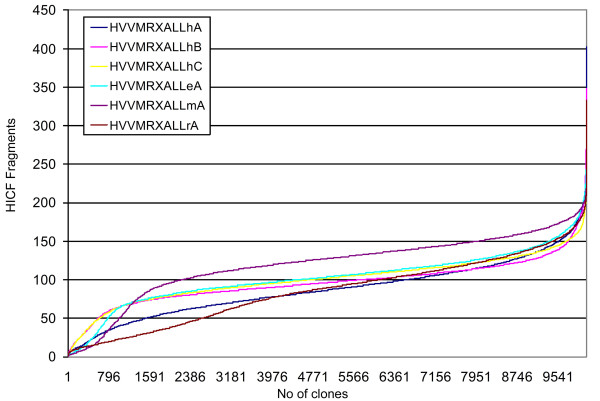
**From all BAC libraries random subsamples between10,279-10,685 clones (Table 2) were fingerprinted (HICF)**. The total number of fragments per analyzed clone was plotted in ascending order for each library. The legend shows the color-coding for the investigated libraries

The HVVMRXALLmA library showed the largest average insert size of 143 Kb with an equal distribution around the mean and the highest average number of fingerprint fragments (Table 1 and 2, Figure 3).

Clones from the HVVMRXALLeA library contained the second largest average insert size of 125 Kb, but insert sizes showed more variation around the mean value as determined by HICF (Figure 3). Libraries HVVMRXALLhA and HVVMRXALLhB contributed clones with medium insert sizes between 97 Kb and 100 Kb. For these two libraries the variation of insert size and average number of fragments around the mean value was more distinct (Table 1, Figure 3). The library HVVMRXALLrA obtained from randomly sheared DNA showed the smallest average insert size of 92 Kb. Each library represented between 2.4 to 6.6-fold the haploid barley genome (Table 1). Together with the previously published BAC library of Yu et al. (2000) [29], more than 25-fold combined haploid genome coverage is available now in BAC libraries of the six-rowed malting barley cultivar Morex (Table 1). The probability to recover any specific sequence of interest is > 99% across all libraries [56].

Irrespective of the BAC cloning method (restriction enzyme, DNA-shearing; see above) the average BAC insert size has a major impact on the contribution to the physical map.

There is a positive relation between the BAC insert size and number of fragments depending on the chosen fingerprinting technique [54,57]. During this study, for the investigated barley libraries, we observed a positive correlation between "insert size" and "number of fragments" as mentioned before (see Table 1, Figure 3). Furthermore, Meyers et al. (2004) [54] investigated the contribution of overall fragment numbers per clone vs reliability of clone overlap at a given suslton score, a key parameter used in FPC (Fingerprint Contig [40]). It was observed that increasing total fragments per clone in turn increases the overlapping BACs at a given Sulston score thus decreasing the occurrence of false-positives. But there is potential fragment size saturation where an increased number of bands does produce false overlaps in a contig assembly [54].

After assembling the BACs into contigs, the Minimal Tiling Path (MTP) selection will be the basis for BAC-by-BAC sequencing. There is a preference of selecting large insert clones [58] which has the advantage that less BACs must be chosen for the MTP and a maximum of sequence information could be obtained from each BAC [59]. But also the risk of a chimeric or contaminated BAC should be kept in mind [58].

For the maize physical map large insert sized BACs were used as "seed" BACs in the maize MTP construction, which provided the highest information content to confirm overlaps between adjacent BACs [60]. For some genome regions large or medium-size clones generated by different methods and or techniques (e.g. BACs from a different BAC library, fosmids) were chosen to fill gaps indicating that depending on the sequence, different type of clones were needed to cover the genome [60]. Therefore the five BAC libraries described in this study provide an optimal resource for whole genome physical mapping of the barley genome with minimal gaps.

### Quality parameters of BAC resources

During the cloning procedure of a BAC library there is a risk of over-representation of organelle DNA which is mixed in various amounts with isolated nuclei in the process of preparing high molecular weight (HMW) DNA. A random clone set of each BAC-library (10,279 -10,685 samples) was investigated by HICF (see above). This also included a BAC clone known to represent the entire chloroplast of cv. Morex [61]. Including this clone into HICF provided a reference fingerprint which then could be compared to all other high-quality BAC fingerprints. At a threshold of higher than 50% identical fragments to the chloroplast control, BAC clones were flagged as originating mainly from chloroplast DNA. The highest percentage of chloroplast-BACs (1.85%) was found in the library HVVMRXALLhC (see Table 3). Medium-level chloroplast-contamination was observed for the libraries HVVMRXALLhB, HVVMRXALLhA and HVVMRXALLrA with 0.92%, 0.78% and 0.45%, respectively. The smallest amount of chloroplast-DNA contamination was observed in HVVMRXALLeA (0.11%) and HVVMRXALLmA (0.07%). Due to the lack of sequenced BAC clones that represent the entire mitochondria of barley, contamination of BAC libraries by mitochondrial DNA was not determined.

**Table 3:** Quality parameter of BAC libraries determined with FPMiner 2.0 software.

BAC library	V	PWC	NC	CC	EW	SHC
HVVMRXALLhA	83.14%	7.28%	2.73%	0.78%	0.23%	6.53%
HVVMRXALLhB	89.43%	4.63%	1.61%	0.92%	0.20%	3.34%
HVVMRXALLhC	87.74%	5.76%	2.09%	1.85%	0.41%	2.83%
HVVMRXALLeA	91.73%	1.44%	1.12%	0.11%	0.36%	5.24%
HVVMRXALLmA	87.46%	4.22%	1.17%	0.07%	1.41%	5.67%
HVVMRXALLrA	80.49%	3.14%	1.01%	0.45%	0.33%	14.58%

V = Valuable clones; PWC = plate-wide contamination; NC = neighboring contamination; CC = chloroplast-contamination; EW = empty wells; SHC = Clones with <30 or >250 fragments, lower (<30) and upper (> 250) border for total number of fragments per clone, which will be exported to FPC (see also material and methods); n.d. = not determined.

During the process of clone picking, plate replicating and re-arraying of clones there is a risk of introducing contaminations between BAC clones even if lab automation is used. Such contaminations maybe observed by fragment pattern identity of neighboring clones within a multi-well plate. The potential neighboring and/or plate-wide contaminations were determined by comparing HICF profiles of the ~10,000 clones fingerprinted for each library. If the overall fragment identity of two clones at neighboring position within one plate or at identical position in subsequent plates of the library is higher than 50%, these clones were flagged. The highest rate of potential neighbor (2.73%) and plate-wide (7.28%) contamination was observed in library HVVMRXALLhA. For this library no values for these two parameters were given by Yu et al., 2000 [29]. During this study we used a copy made several years ago which in between has been extensively used for other purposes. Therefore we cannot rule out that contaminations introduced over time during plate handling.

Potential neighbor contaminations were found to be in the range between 1.01% and 2.09% for the other libraries and plate-wide contaminations were as high as 1.44% to 5.76% (Table 3).

Contaminated clones may be identified also by overall fragment number in HICF analysis. If a single glycerol stock would contain two different BACs of similar size, HICF analysis would indicate twice the number of fragments as compared to a normal clone of the same library.

Besides contaminations and clones with too few or too many fingerprint fragments, empty vector clones or non-viable clones can compromise the quality of a BAC library since such "empty" wells in BAC-library plates increases the preparation costs and increases the need for larger number of clones to be processed in fingerprinting if used for physical map construction. A very small fraction of "empty" wells was found for all libraries (> 0.35%-2.9%, Table 3).

The number of fragments after HICF, is an exclusion parameter for clones during systematic physical map construction. In contrast, BAC clones with very small inserts would provide too little information from HICF for being valuable for physical mapping. Clones with less than 30 fragments would have a very small overlap to other clones and would therefore most likely stay as singletons or overlaps would remain uncertain. If a high number of small inserts were obtained in a library, size selection of HMW DNA before cloning would probably be inefficient, because small fragments tend to co-migrate with larger fragments in highly concentrated samples [62] and sheared large DNA fragments are far less efficient to be cloned. Therefore both cases - too many and too few fragments compared to the average - would need to be filtered in a systematic physical mapping project. In this study the average number of fragments over all libraries was 98.6. The percentage of clones which fell into the range of <30 and >250 fragments varied among the libraries (Table 3). It cannot be ruled out that large BAC clones containing large numbers of highly conserved tandem repeats, centromeric and telomeric repetitive sequences could potentially produce less than 30 fragments by HICF (Cheng-cang Wu, unpublished data). This may explain partly the highest percentage (SHC: 14.58% in Figure 3) of clones with <30 or >250 fragments found in the sheared BAC library (HVVMRXALLrA) which is expected to cover regions underrepresented in libraries obtained by partial digest of HMW DNA. However, further experimentation is required to test this hypothesis.

### Experimental validation of genome representation

Theoretical assumptions about genome coverage of newly developed BAC libraries based on clone numbers and average insert sizes of sample clones remains uncertain since such analyses do not reveal potential redundancy in libraries introduced during the cloning procedure (i.e. overgrowth of transformation assays). Therefore, high density colony arrays of all libraries were screened with a set of single- or low-copy gene probes (see additional file 3 and 4). The library HVVMRXALLhA was excluded from this screening, since it was already intensively characterized in previous studies [29,63,64].

**Figure 4:**
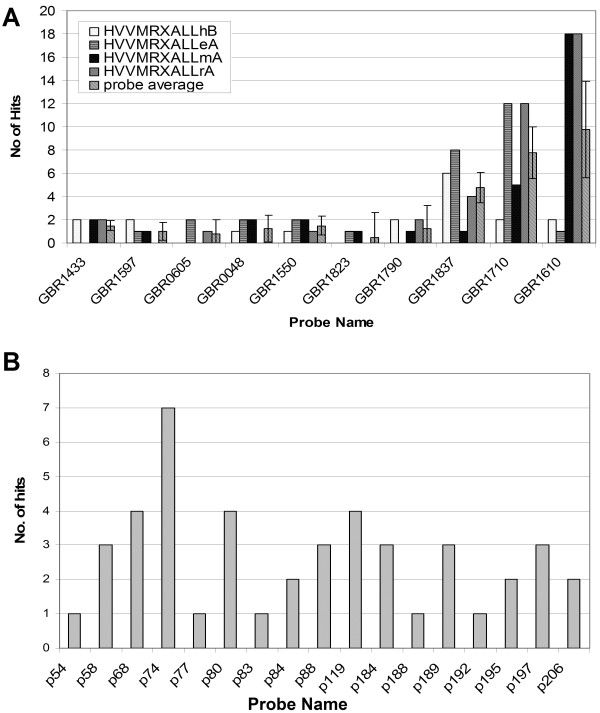
**Hybridization results of EST-derived probes on different BAC-filters**. Probe details are provided in additional file 2. **A**: Hybridization with GBR-probes on 5 BAC libraries. The probe average including the standard deviation is given in the last column **B**: Hybridization result on HVVMRXALLhC library with 17 EST-derived probes assigned to chromosome 3H

The libraries HVVMRXALLeA, HVVMRXALLmA, HVVMRXALLrA, and HVVMRXALLhB were hybridized with ten RFLP-markers [65]. These markers were tested before by Southern analysis to represent single or low-copy sequences (data not shown) and were known to be distributed on barley chromosomes 2H, 3H, 5H - 7H (additional file 3). A single colony filter per library comprising 55,296 clones was probed (additional file 1). On average, 1 to 7 BAC addresses could be identified (Figure 4A).

None of these four libraries showed any significant pattern of library amplification since the number of positive signals obtained correlated well with the expected number (Figure 4A, additional file 4). All clones identified by screening of high density colony filters were analyzed by HICF and for six of the ten GBR probes (GBR0048, GBR0605, GBR1550, GBR1597, GBR1790, GBR1823) all clones assembled into single contigs confirming the single-copy character of the probes. BACs identified by GBR1433, GBR1837, GBR1710 and GBR1610 assembled in two, three, nine or even ten contigs, respectively. Given the single copy nature of the probes in previous Southern analysis, the finding of two or three independent contigs per single copy probe may be explained by too little overlap of positive BAC clones in the area carrying the respective genes thus not allowing FPC to build single contigs. In the cases of larger number of contigs it is likely that such markers cross-hybridized to more paralogous genes than could be expected from the previous Southern evaluation (data not shown).

One obvious observation was that there are more hits than average for single-copy probes in the library HVVMRXALLhA; but the positive clone numbers are consistently corresponding to contig numbers (probably paralogous gene numbers) only in the sheared BAC library HVVMRXALLrA for all three low-copy probes: GBR1837, GBR1710, and GBR1610 (Figure 4A). The un-biasness of the sheared BAC library compared to the partial digestion BAC libraries may be apparent by screening more DNA probes including repetitive sequences.

The entire HVVMRXALLhC library was screened with a set of seventeen wheat EST-derived probes previously mapped to wheat chromosome 3D. Because wheat and barley genomes are closely related, probes from one species can easily be used against genomic filter of the other. These probes were first hybridized on wheat nulli-tetrasomic lines in order to verify the 3D location and afterwards on the HVVMRXALLhC-filters to identify the syntenic barley regions. In total 8 of 17 EST-Markers (p58, p67, p77, p84, p88, p119, p188, p195) gave exactly the expected number of BACs (coverage of the filter set = 3.4 x for the entire HVVMRXALLhC-library). The remaining probes revealed at least a single BAC address. On average the probes revealed 2.8 BAC addresses (Figure 4B). The copy number of the probes was calculated in wheat nulli-tetrasomic lines and therefore could differ in the barley genome due to sequence variations.

## Conclusion

In this paper we report on the development and characterization of a set of five new publicly available BAC libraries of barley cultivar Morex - a cultivar selected by the International Barley Sequencing Consortium as reference genotype for genome sequencing [21]. Altogether the libraries represent >25-fold the haploid genome of barley. The libraries were generated from HMW DNA partially digested with different restriction endonucleases or mechanical shearing in order to reduce the risk of genome regions being under-represented in the libraries - an aspect which would interfere with the aim of developing a physical map of the entire barley genome. Based on the analyzed quality parameters and the obtained experimental evidences it can be concluded that the new libraries (1) represent a comprehensive reagent for gene discovery and (11) can be utilized for developing a generic physical map of barley.

## Authors' contributions

NS conceived the project in collaboration with AG and PL. DS and RA performed the fingerprinting, barley probe hybridization and data analysis steps. BS constructed the HVVMRXALLhB and HVVMRXALLhC libraries, MA and CS constructed the HVVMRXALLeA library. PJ constructed the HVVMRXALLmA library and library HVVMRXALLrA was constucted by CCW. DF performed the hybridisation of wheat probes to BAC membranes. The manuscript was written by RA, DS, and NS. All authors read and approved the manuscript.

## Supplementary Material

Additional file 1 **Overview of available high density filter resources for all BAC libraries and used filter set for validation**. Click here for file

Additional file 2 Detailed information of probes Click here for file

Additional file 3 **Detailed hybridization results of EST-derived probes on BAC-filters**. Click here for file

Additional file 4 **After hybridizing 10 RFLP probes to the HVVMRXALLhB, HVVMRXALLmA, HVVMRXALLeA and HVVMRXALLrA library the copy number was recalculated according to the contig results of an assembly with positives clones**. Click here for file
