# Supplementary material for: BAC library resources for map-based cloning and physical map construction in barley (Hordeum vulgare L.)
Source: BMC Genomics. 2011 May 19;12:247. doi: 10.1186/1471-2164-12-247 (PMC3224359; doi:10.1186/1471-2164-12-247)
Supplement: Additional file 1 — Overview of available high density filter resources for all BAC libraries and used filter set for validation. [file 1471-2164-12-247-S1.DOC]

**Additional file 1:** Overview of available high density filter resources for all BAC libraries and used filter set for validation.

n.u.= not used

| BAC library | Spotting pattern on filter | Total No. of plates per BAC-library | Plate no. on filter used for hybridization | ordering information |
| --- | --- | --- | --- | --- |
| HVVMRXALLhA | 4x4 | 816 | n.u. | <http://www.genome.clemson.edu/online_orders> |
| HVVMRXALLhB | 7x7 | 300 | 50-180 | <http://cnrgv.toulouse.inra.fr/en/library/barley> |
| HVVMRXALLhC | 4x4 | 480 | 1-480 |  |
| HVVMRXALLeA | 7x7 | 384 | 1-144 | <http://www.genome.clemson.edu/online_orders> |
| HVVMRXALLmA | 7x7 | 528 | 1-144 | <http://cnrgv.toulouse.inra.fr/en/library/barley> |
| HVVMRXALLrA | 7x7 | 678 | 1-144 | <http://cnrgv.toulouse.inra.fr/en/library/barley> |
|  |  |  |  |  |
