# Supplementary material for: BAC library resources for map-based cloning and physical map construction in barley (Hordeum vulgare L.)
Source: BMC Genomics. 2011 May 19;12:247. doi: 10.1186/1471-2164-12-247 (PMC3224359; doi:10.1186/1471-2164-12-247)
Supplement: Additional file 2 — Detailed information of probes [file 1471-2164-12-247-S2.DOC]

**Additional file 2**: Detailed information of probes.

s = single copy; s-l = Single – low copy; l = low copy

| Probe | Unigene U35^1^ | Primer F 5' -3' | Primer R 3'-5' | Copy No. | EST^1^ | Chr | Position in cM* |
| --- | --- | --- | --- | --- | --- | --- | --- |
| GBR1433 | 42367 | CGCAGGGCCTTTATGTGATGAAC | GCTCTCGGCAACAGGCAAGTAAG | s | HT14I11 | 3H | 75.2 |
| GBR1597 | 16987 | AGGGCTGCATGAACAATCTGAC | TCTGCGACCATCTTTCCTGACTA | s-l | HO12J24 | 6H | 47.2 |
| GBR0605 | 22947 | ATGGAGCAGCGGCAAGGCAAGAT | CTCAGATGGCACGGCGACAGC | s-l | HT02J04 | 7H | 10.4 |
| GBR0048 | 39766 | GCCATCTCCATCAGCACCTAT | ATGGCACCGTATTGTCTTTCA | s-l | HO06M04 | 3H | 106.2 |
| GBR1550 | 22063 | GGTAAAGGCAGCCCCGAGTTCAG | GGCGCCAATGTACCCAAGTGC | s-l | HY02E13 | 2H | 41.1 |
| GBR1823 | 16537 | GCCTCCCGTGCCCTTCTA | CACCGATTTTCCAGCCACTACAG | s-l | HD08A12 | 7H | 4.7 |
| GBR1790 | 802 | CCTCGGTATGGGTGTTTCTGGA | AGTTTCGCCGCTGCTGTAGTCAC | s-l | HW04J10 | 3H | 72.9 |
| GBR1837 | 38758 | CGCGCATCGATGAGTGGAAACC | ATGCGATCGGAGAAAGGAAGACTG | s-l | Hi06M17 | 6H | 71.3 |
| GBR1710 | 13831 | GCTGGGCTGCTGGAGAAGAAATA | GTGGCGGAGAAGACGGTGTAGAC | s-l | HA02B08 | 5H | 123.5 |
| GBR1610 | 17730 | GCATTTGGGAGGCTTCTGATTCT | GTGACCTGCCGGCTTGTGATAAA | s-l | HV06G03 | 7H | 68.3 |
|  |  |  |  |  | Wheat EST+ |  |  |
| p54 | 1166 | AGAATGCCTCGCTACGATGA | GGAACCCCTTTAGCAAACTCAAC | s-l | TA51544_4565 |  |  |
| p58 | 5623 | CACTTGACATTTGATGAAACTCTCTT | TGGTAAATCTCAGGTTCAGAAGACT | s-l | BE498698 |  |  |
| p67 | 27225 | AAGATTCTCCTGATGATGTTGAG | TAGGACTTGAATTCCACATTTGC | s | TA67203_4565 |  |  |
| p68 | 18319 | ATTGACGTCGGTGGTGAGA | CTTTCTTGATCGCCTGCTTG | s-l | TA90323_4565 |  |  |
| p74 | 27980 | CCTCGGACCAAACTTGTTGA | ATTCCACAACCAAAGCAAGG | s | TA51777_4565 |  |  |
| p77 | 1198 | GCCCCACTTCAACAACATTC | GGACAGGCCATCTTTGACTC | s-l | TA96588_4565 |  |  |
| p80 | 16014 | CGCTCAAGACGCAGTACAAG | ATCATGTCGATAACCTCCATCAC | s | TA70179_4565 |  |  |
| p83 | 1283 | TGCTTGGATTCAGGCTTCTT | CCATTCACTTGGTTTTCTGGA | s | TA57973_4565 |  |  |
| p84 | 18745 | ATTTTGGGGCTTGGAGATTT | AACTGACCTGCAAGGACCAC | s | TA53956_4565 |  |  |
| p88 | 8584 | CTGCGGCGTGAAGTAGAGAT | AACAGACCAGCCGAAGTCAG | s | TA79548_4565 |  |  |
| p119 | 24363 | GTTTGCTTGAGCGATTTCCT | ATCCTCGCCACCTTCTTTTT | s | TA67759_4565 |  |  |
| p184 | 19100 | CATGTATTGGGTGGTGCTTG | GCAGAGCATGAAAAGGAAGC | s-l | TA58417_4565 |  |  |
| p188 | 6900 | GGATGCTCTTGAGTCGTGAA | ACCACAGGTAACCTCGCAAC | s | TA77637_4565 |  |  |
| p189 | 4247 | TCCAAAAACAGTGTGGCAAA | GCCAAGTCGATCCAAGTGTT | s | BE500151 |  |  |
| p192 | 21785 | GGCAAGCAAAGACAACGAC | CTCGTCGTCCTCGTACACC | s | CJ518569 |  |  |
| p195 | 17856 | GGCCAAGAAAGATAGAAGAAAGAA | CACATCAAGGGTCGGATTTG | l | TA109799_4565 |  |  |
| p197 | 24307 | GTGCGGATGGTGATGTCC | CGCATCTTATGTTCTCATCAGG | s | TA71464_4565 |  |  |

| * Stein et al., 2007; + <http://plantta.jcvi.org/>; ^1^ http://www.harvest-web.org/ |
| --- |
