# Supplementary material for: BAC library resources for map-based cloning and physical map construction in barley (Hordeum vulgare L.)
Source: BMC Genomics. 2011 May 19;12:247. doi: 10.1186/1471-2164-12-247 (PMC3224359; doi:10.1186/1471-2164-12-247)
Supplement: Additional file 3 — Detailed hybridization results of EST-derived probes on BAC-filters. [file 1471-2164-12-247-S3.DOC]

| probe | gene  copy | HVVMRXALLhB | HVVMRXALLeA | HVVMRXALLmA | HVVMRXALLrA |
| --- | --- | --- | --- | --- | --- |
| GBR1433 | s | 2 | 0 | 2 | 2 |
| GBR1597 | s-l | 2 | 1 | 1 | 0 |
| GBR0605 | s-l | 0 | 2 | 0 | 1 |
| GBR0048 | s-l | 1 | 2 | 2 | 0 |
| GBR1550 | s-l | 1 | 2 | 2 | 1 |
| GBR1823 | s-l | 0 | 1 | 1 | 0 |
| GBR1790 | s-l | 2 | 0 | 1 | 2 |
| GBR1837 | s-l | 6 | 8 | 1 | 4 |
| GBR1710 | s-l | 2 | 12 | 5 | 12 |
| GBR1610 | s-l | 2 | 1 | 18 | 18 |
| Avg hits in library | | 1.8 | 2.9 | 3.3 | 4 |
| Coverage filter set | | 1.7 | 1.4 | 1.6 | 1 |
| p-value^§^ |  | 0.8554 | 0.258 | 0.3399 | 0.0862 |
|  |  | HVVMRXALLhC | |  |  |
| p54 | s-l | 1 |  |  |  |
| p58 | s-l | 3 |  |  |  |
| p68 | s | 4 |  |  |  |
| p74 | s-l | 7 |  |  |  |
| p77 | s | 1 |  |  |  |
| p80 | s-l | 4 |  |  |  |
| p83 | s | 1 |  |  |  |
| p84 | s | 2 |  |  |  |
| p88 | s | 3 |  |  |  |
| p119 | s | 4 |  |  |  |
| p184 | s-l | 3 |  |  |  |
| p188 | s | 1 |  |  |  |
| p189 | s | 3 |  |  |  |
| p192 | s | 1 |  |  |  |
| p195 | s | 2 |  |  |  |
| p197 | l | 3 |  |  |  |
| p206 | s | 2 |  |  |  |
| Average hits in library | | 2.8 |  |  |  |
| Coverage filter set | | 3.4 |  |  |  |
| p-value^§^ |  | 0.1445 |  |  |  |

**Additional file 3**: Detailed hybridization results of EST-derived probes on BAC-filters.

s=1 band for each enzyme in Morex genotype; s-l=1 or 2 bands; l= 3 bands and more; ^§^ = after performing student t test
