# Supplementary material for: BAC library resources for map-based cloning and physical map construction in barley (Hordeum vulgare L.)
Source: BMC Genomics. 2011 May 19;12:247. doi: 10.1186/1471-2164-12-247 (PMC3224359; doi:10.1186/1471-2164-12-247)
Supplement: Additional file 4 — After hybridizing 10 RFLP probes to the HVVMRXALLhB, HVVMRXALLmA, HVVMRXALLeA and HVVMRXALLrA library the copy number was recalculated according to the contig results of an assembly with positives clones. [file 1471-2164-12-247-S4.DOC]

**Additional file 4**: After hybridizing 10 RFLP probes to the HVVMRXALLhB, HVVMRXALLmA, HVVMRXALLeA and HVVMRXALLrA library the copy number was recalculated according to the contig results of an assembly with positives clones.

+ = expected copy-number after hybridization to southern blots; ^§^ = after performing a student t-

test.

| Probe | No of contigs | No of hits | Average no  hits per library | Expected copy number | p-value |
| --- | --- | --- | --- | --- | --- |
| GBR0048 | 1 | 5 | 1.3 | 1-2 | 0.391 |
| GBR0605 | 1 | 3 | 0.8 | 1-2 | 1.000 |
| GBR1550 | 1 | 6 | 1.5 | 1 | 0.638 |
| GBR1597 | 1 | 4 | 1.0 | 1-2 | 0.638 |
| GBR1790 | 1 | 5 | 1.3 | 1-2 | 0.182 |
| GBR1823 | 1 | 2 | 0.5 | 1-2 | 0.182 |
| GBR1433 | 2 | 6 | 1.5 | 1-2 | 0.638 |
| GBR1837 | 4 | 19 | 4.8 | 1-2 | 0.087 |
| GBR1710 | 9 | 31 | 7.8 | 1-2 | 0.076 |
| GBR1610 | 10 | 39 | 9.8 | 1-2 | 0.164 |
